# Pesticides, an urgent challenge to global environmental health and planetary boundaries

**DOI:** 10.3389/ftox.2025.1656297

**Published:** 2025-10-03

**Authors:** Laura N. Vandenberg, Elise J. Pierce, Rachel M. Arsenault

**Affiliations:** Department of Environmental Health Sciences, School of Public Health and Health Sciences, University of Massachusetts Amherst, Amherst, MA, United States

**Keywords:** sustainability, endocrine disrupting chemical, insecticide, herbicide, equity, justice, disparities

## Abstract

There is increasing evidence that pesticides act as endocrine disruptors, developmental toxicants, and reproductive toxicants. In this review, we describe several global challenges associated with pesticide production and use that put the health of human and wildlife populations at risk. These include: (1) the global production and use of pesticides is high, leading to increasing rates of release into the environment; (2) exposures to non-target species (including humans) are well documented, and pesticides often have adverse effects on these species; (3) pesticides, and especially those that are persistent organic pollutants, do not stay where they are used, contributing to ecosystem pollution far from their intended areas of application; (4) climate change can exacerbate the use of pesticides; and (5) social determinants of health (race/ethnicity, sex, and occupation) influence pesticide exposures and the adverse effects associated with these exposures. In 2009, the concept of planetary boundaries was introduced as a framework to evaluate how human actions impact earth systems. The planetary boundaries were based on a shared understanding that human activities have significant and sometimes irreversible effects on key aspects of environmental health. When considering the global impact of pesticides, these products can disrupt several planetary boundaries including biogeochemical cycles, biosphere integrity (e.g., measures of biodiversity), and the availability of clean freshwater, but the greatest challenge posed by pesticides is the “novel entities” boundary (i.e., the introduction of synthetic chemicals and materials into the environment). The planetary boundaries framework makes clear that failure to act against the most concerning chemicals, including pesticides, ultimately puts the survival of human populations at risk.

## Background

### Pesticides: a feature of modern living

Pesticides are biologically active chemicals used for the control of organisms that are considered pests including plants, insects, rodents, fungi, bacteria, and microbes. In some of the earliest human civilizations, elements (e.g., sulfur, arsenic, mercury, lead) and extracts from plants were used in crop production because of their ability to control pests (Kughur, 2012). Since the 1940s, with the chemical revolution that accompanied World War II, many pesticides were designed to target specific molecular pathways and cellular receptors in target species. Several categories of plant-derived pesticides, utilizing chemistry found naturally in botanicals, have now been developed (Kamrin, 1997). Pesticides have become vital to crop production and are an integral part of food production. About the herbicide glyphosate, for example, some environmental policy experts have written, “there is little doubt that [its] use massively boosts agricultural productivity, at least on the short term” (Dorlach and Gunasekara, 2023). The abrupt ban of the use of synthetic agrochemicals (including both pesticides and chemical fertilizers) in Sri Lanka in 2021 was disastrous for production of critical crops including rice and tea, contributing to an increase in measures of food insecurity (Drechsel et al., 2025). Although it was not possible to determine the impact of the ban on pesticides specifically (because chemical fertilizers were concurrently banned, and the country also experienced a severe drought during the growing season), a survey of farmers indicated that many reported an increased problem with both weeds and insects.

Yet, there are issues with some of the claims that have been made about the benefits of pesticides on crop production. In several regions of the world, crop yields have not improved in spite of the increased use of pesticides, and some yields may have even declined (Ray et al., 2012). Atrazine is a high production volume herbicide that has been described as essential for the production of corn (Mitchell, 2011). However, when atrazine use was restricted in several countries in the European Union, there was no effect on corn crop productivity in these locales (Ackerman, 2007). A 2014 analysis by agricultural economists concluded that elimination of the use of atrazine in the United States would lead to more than US$1.5B in additional revenues for corn growers, even if other pesticides were substituted for atrazine (Ackerman et al., 2014). Similarly, a regional ban of 14 agricultural pesticides that were considered highly hazardous in the Indian state of Kerala resulted in no evidence for reduced yield of eight crops in the year the ban was initiated (2011) or the following year (Sethi et al., 2022). These eight pesticides were selectively banned because they had contributed to thousands of poisonings that led to deaths. Sadly, suicide by pesticide poisoning disproportionately affects countries that utilize highly hazardous pesticides including Sri Lanka, Bangladesh, and other parts of India (Gunnell et al., 2007; Chowdhury et al., 2018; Bonvoisin et al., 2020).

When specific pesticides are phased out of use (due to regulation or loss of efficacy as resistant pests arise), typically one of two options is selected by agricultural producers. The first is the adoption of other pesticides; this has been observed in many global jurisdictions as herbicides like atrazine and glyphosate are used less frequently, and replacements such as dicamba and 2,4-D are used in their place (Wechsler et al., 2019). These replacements are especially concerning because of evidence that they may be even more toxic than the herbicides they are replacing. The second approach is to utilize integrated pest and pesticide management strategies (Peshin and Zhang, 2014), which can include a shift to less-toxic chemical pesticides, better use of transgenic crops, use of improved cultivation techniques, protection and enhancement of beneficial organisms, as well as non-chemical methods to control pests (Barzman et al., 2015).

Pesticides are not only used in the protection of crops, but also to control species that transmit deadly diseases that impact the health of human and wildlife populations such as West Nile virus, malaria, and Dengue fever, among others (Whitford, 2002). Thus, it has been argued that pesticides increase quality of life (Whitford et al., 2006) by reducing the impact of infectious diseases that, uncontrolled, would have morbidity and mortality rates potentially affecting more than a billion people (Hay et al., 2004).

**FIGURE 1:**
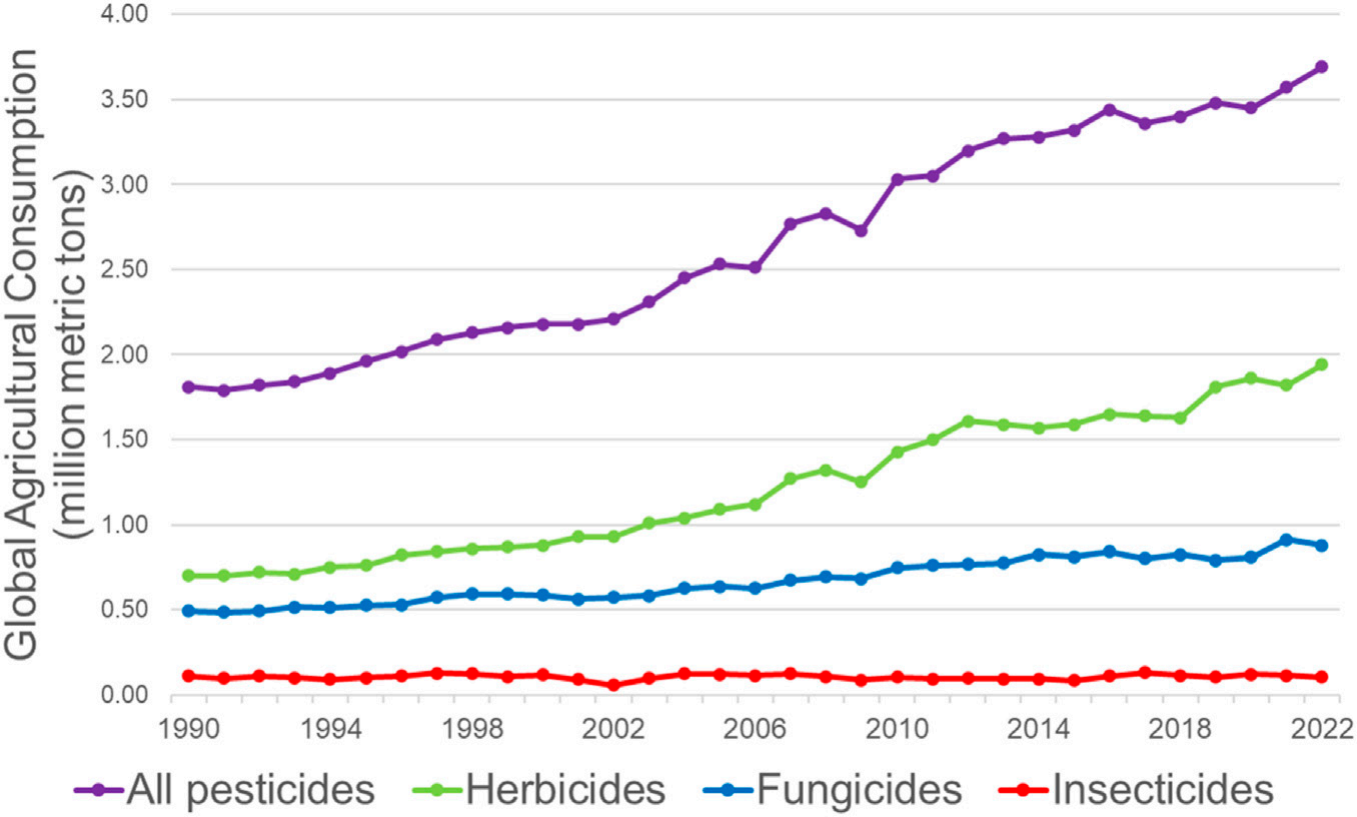
Global pesticide use increased significantly from 1990 through 2022. Data from the FAO illustrates the steady increase in the volume of pesticides used for agricultural purposes globally over a period of 3 decades. Interestingly, much of the increase in total pesticides used can be attributed to the increase in use of herbicides, whereas the increase in fungicides (and other biocides) was more modest and insecticide use remained largely steady. Data from: (Faostat Analytical Brief, 2022).

As global annual agricultural use of pesticides increased from 1.81 million metric tons in 1990 to 3.69 million metric tons in 2022 (a 104% increase over 3 decades, see Figure 1), there are increasing concerns that pesticides are contributing to negative health outcomes in exposed individuals, including those that are exposed occupationally as well as the general public who consume agricultural products (Petit and Vuillerme, 2025). In this review, we discuss some of the earliest evidence that many pesticides are hormonally active, and thus are endocrine disrupting chemicals (EDCs), developmental toxicants, and reproductive toxicants. We examine several reasons why pesticides create global challenges, and examine pesticides using the framework of planetary boundaries, which evaluates how human activities impact earth systems and prevent the earth’s biophysical systems from being maintained sustainably.

### Many pesticides are endocrine disrupting chemicals, developmental toxicants, or reproductive toxicants

In the early 1990s, a report from the US National Academy of Sciences documented the extent that pesticides were found in the diets of children (Council, 1993). Further concern was raised because of evidence that many pesticides had unintended effects on the endocrine systems of wildlife and humans (Colborn et al., 1993). The 1991 Wingspread Conference examined the consequences of pesticides, as well as other environmental chemicals and hormonally active pharmaceuticals. Researchers at this conference coined the term “endocrine disruptor” to describe chemicals that can bind to hormone receptors or alter some other aspect of hormone action to disturb the health of the individual (Colborn et al., 1993; Colborn and Clement, 1992). Even at that early date, there was clear evidence that hormonally active pesticides were disrupting development and reproduction of species including wildlife and humans.

This newfound scientific attention in the 1990s led the US Congress and several US regulatory agencies to acknowledge that many pesticides could induce harmful effects by mimicking or blocking the actions of sex hormones; several were also determined to be developmental and reproductive toxicants (Colborn et al., 1995; Krimsky, 2003). In 1996, the US Congress signed into law the Food Quality Protection Act (Congress, 1996) which required the US EPA to create a screening program to evaluate pesticides for several endocrine disrupting properties. In response to this law, the EPA assembled a scientific advisory committee (EDSTAC, 1998) which recommended the creation of a two-tiered endocrine disruptor screening program (EDSP) to identify chemicals that bind to the androgen, estrogen and thyroid hormone receptors (EPA, 2013). Unfortunately, as recently described (Maffini and Vandenberg, 2022), more than 1300 chemicals were identified as “high priority” for screening by the EPA because of their use in pesticides, but by 2022, fewer than 100 had been screened through the first tier of the EDSP, and none had been tested in Tier 2 (Oig, 2021).

In the European Union, early efforts undertaken to address the problem of EDCs were launched in 1999 with the “European Strategy on EDCs” (Kassotis et al., 2020). Although it was not specifically focused on pesticides, the strategy included significant funding for EDC research, and paved the road for EU laws on pesticides and biocides. However, it took until 2009 for the EU “Plant Protection Products Regulation 1107/2009” to be finalized, which specifically focused on regulating agricultural pesticides with endocrine disrupting properties. This regulation, together with the 2012 EU Biocides regulation, disallowed the authorization of substances identified as EDCs. Based on these laws, several pesticides have been banned from use in the EU. However, the EU Strategy has been criticized by public health advocates for being too slow, for having “blind spots” for some features of EDCs, and for not treating the problem of EDCs with sufficient gravity (van Vliet and Jensen, 2013).

Beyond the US and EU, the United Nations Environment Programme has acknowledged the global challenge of EDCs (Bergman et al., 2013) and the Organization for Economic Cooperation and Development (OECD) has led efforts to develop globally harmonized methods to test chemicals for some kinds of endocrine disrupting properties. However, these organizations also acknowledge that governments worldwide have very different approaches to the regulation of chemicals, including pesticides, which poses significant challenges to their testing and to regulatory oversight (Kassotis et al., 2020).

Since the earliest policy and regulatory responses to the question of agrochemicals with endocrine disrupting properties, an increasing understanding of the endocrine system and the principles of endocrinology has allowed for more advanced knowledge of the mechanisms by which pesticides with endocrine disrupting properties affect the health of individuals and populations (Zoeller et al., 2012; Vandenberg et al., 2013; Gore et al., 2015; Schug et al., 2013). There has been debate amongst scientists and regulators around the world about the best ways to define (and thus identify) EDCs (Zoeller et al., 2014). However, the development of ‘key characteristics’ of carcinogens, EDCs, and male and female reproductive toxicants have provided a useful framework by which chemicals can be evaluated and the evidence for their toxicity clearly assembled (La Merrill et al., 2020; Luderer et al., 2019; Arzuaga et al., 2019). Such approaches have started to be used for the evaluation of pesticides including dichlorodiphenyltrichloroethane (DDT), endosulfan, atrazine, and glyphosate (Calaf et al., 2020; Vandenberg et al., 2020; Muñoz et al., 2021; Rana et al., 2023).

DDT is perhaps one of the most well-studied pesticides, and its effects have been felt globally. This insecticide targets voltage-gated sodium channel proteins found in the membranes of nerves and neurons, and binding of DDT to these proteins disrupts the normal transmission of nerve impulses, leading to seizures or paralysis, followed by death (Davies et al., 2007). In addition to this mechanism of action, DDT has been shown to be a non-genotoxic carcinogen, an activator of the constitutive androstane receptor, an inhibitor of gap junctions, and an inducer of oxidative stress (Harada et al., 2016). DDT and its metabolites are known EDCs, possessing both estrogen receptor agonist and antagonist activities (Vandenberg et al., 2020). Furthermore, dozens of epidemiology and wildlife studies have shown associations between DDT exposures adverse health outcomes including cancer, altered immune system functions, disruptions to reproductive health including alterations to the sperm epigenome which could impact future generations, and altered neurological development in humans (World Health Organization, 2003; Marlatt et al., 2022; Lismer et al., 2024).

Systematic reviews have provided evidence that several pesticides are developmental and reproductive toxicants. For example, occupational exposure to pesticides (defined broadly) has been shown to be associated with measures of male reproductive toxicity, including adverse effects on measures of sperm motility and DNA integrity (Knapke et al., 2022). Pregnant women exposed to pesticides (defined broadly) were also shown to be at increased risk for spontaneous abortion (Albadrani et al., 2024). Systematic reviews that examine specific pesticides have also provided evidence that these chemicals alter reproductive outcomes in exposed animals and/or human populations. For example, the fungicide mancozeb has been shown to alter fertility outcomes in multiple species of laboratory animal (Runkle et al., 2017); the fungicide vinclozolin alters sperm motility, sperm count, and epididymal weight in exposed rodents (Feijó et al., 2021); and the insecticides malathion and diazinon are male reproductive toxicants that damage the Leydig cells in the testis, decreasing the production of androgens and reducing sperm quality in rodents (Delorenzi Schons and Leite, 2023). Furthermore, meta-analyses have revealed an increased risk of breast cancer in women associated with exposures to the insecticide hexachlorocyclohexane (Liu et al., 2023). Evidence for other forms of toxicity (e.g., neurotoxicity) has also been assembled for pesticides like paraquat and chlorpyrifos (Vaccari et al., 2019; Coleman et al., 2025).

Controlled laboratory experiments with model organisms have generated evidence that animals are affected by low dose exposures to pesticides and other environmental chemicals, even when such chemicals are administered to animals below the doses that are used to generate toxicological no-observed-adverse-effect-levels (NOAEL) (Bergman et al., 2013; Zoeller et al., 2012; Gore et al., 2015; Vandenberg et al., 2020; Vandenberg, 2014; Hill et al., 2018; Vandenberg, 2019; Diamanti-Kandarakis et al., 2009; Kortenkamp et al., 2011). More specifically, many of the highest volume pesticides have been shown to have an endocrine mode of action, and many also affect development and/or the reproductive health of animals exposed in controlled laboratory settings (see Table 1). Although risk assessments are required for all pesticides (at least in the United States and EU), hundreds of studies have demonstrated associations between pesticide exposures and adverse health effects in human populations, even when such exposures are low (Gore et al., 2015; Stillerman et al., 2008; Crain et al., 2008; Skakkebaek et al., 2016; Kahn and Trasande, 2018; Wan et al., 2021; Mendes et al., 2021; Rocha et al., 2021; Kahn et al., 2021; Fernández-Martínez et al., 2020; Ribeiro et al., 2020; Fu et al., 2020; Bliatka et al., 2020; Nelson et al., 2020; Rivollier et al., 2019; Wen et al., 2019; Ghassabian et al., 2022). Many environmental epidemiology studies (almost exclusively focused on non-occupationally exposed individuals) have shown associations between exposures to pesticides and adverse health outcomes, including effects on neurobehaviors in children (Thistle et al., 2022), metabolic syndrome (Lamat et al., 2022), risk of cancers (Rossides et al., 2022), and other serious health effects. These outcomes in exposed human populations suggest that the approaches used to evaluate chemical hazards are insufficient to identify “safe” levels of exposure for the general population (Vandenberg, 2019; Vandenberg, 2021).

**TABLE 1:** Examples of high volume pesticides and some of their known endocrine modes of action.

Chemical	Use	Annual production volume	Endocrine mode of action
Glyphosate	Herbicide	136,000 metric tons (United States alone)	There is evidence from a human population cohort that glyphosate-based herbicides are associated with altered anogenital distance, a marker of androgen action (Amreen et al., 2025)
Atrazine	Herbicide	64,000–82,000 metric tons (global)	Evidence from across the animal kingdom that atrazine increases aromatase, ultimately increasing estrogen synthesis (Vandenberg et al., 2012)
malathion	insecticide	6800 metric tons (United States alone)	Evidence that malathion decreases testosterone synthesis (Erthal-Michelato et al., 2024)
cypermethrin	insecticide	450 metric tons (United States alone)	Evidence that cypermethrin alters steroidogenesis and spermatogenesis in exposed rodents (Irani et al., 2022)
metolachlor	herbicide	136,000 metric tons (global)	Evidence that metolachlor alters steroidogenesis and interferes with the hypothalamic-pituitary-testis and hypothalamic-pituitary-adrenal axis in zebrafish (Quintaneiro et al., 2017)
β-hexachlorocyclohexane (β-HCH) also known as lindane	insecticide	5.4 million metric tons (global)	Cell-based assays indicate that β-HCH has estrogenic activity, inducing proliferation of breast cell lines and increasing the expression of estrogen-responsive genes (Silva et al., 2010)
paraquat	herbicide	4500 metric tons (United States alone)	Paraquat has been shown to alter thyroid hormone levels in animals models, and is associated with increased T3, T4 and thyroid stimulating hormone in farmers that use the pesticide (Kongtip et al., 2019)
chlorpyrifos	insecticide	45,000 metric tons (global)	Systematic reviews have revealed that chlorpyrifos alters sex-dependent hypothalamic neuroendocrine pathways in rodents (Venerosi et al., 2012)
endosulfan	insecticide	12,000 metric tons (global)	Cell-based assays have shown that endosulfan has estrogenic activity and can promote estrogen-responsive cells (Soto et al., 1994)
2, 4-D	herbicide	21,000 metric tons (United States alone)	Studies in model organisms including *C. elegans* reveal that 2,4-D has estrogenic activity and exposures can decrease brood size, alter vitellogenin, and shift the expression of genes involved in reproduction (Moya et al., 2022)
dicamba	herbicide	18,000 metric tons (global)	Fish exposed to dicamba have disruptions to spermatogenesis and ovarian degeneration and increased circulation of estradiol (Zhu et al., 2015)

### Global challenges associated with pesticide use

The production of pesticides has been on an upward trajectory for several decades, leading to increasing rates of release into the environment. In this section, we describe how this increasing production of pesticides contributes to exposures (and effects) in non-target species (including humans), especially because pesticides do not stay where they are used.

#### Pesticides are used in high quantities, contributing to widespread human and wildlife exposures

In her 1962 book *Silent spring*, Rachel Carson described the environmental harm caused by the indiscriminate use of pesticides to control insect populations (Carson, 1987). Beginning in the 1930s and 1940s, pesticides like DDT were used as an effective and efficient approach to control insects and protect crops and livestock, and as a malaria preventative measure by targeting mosquitoes (Rattner, 2009). Carson’s book not only revealed the extensive contamination of wildlife populations such as apex predators and birds of prey, she also documented the widespread exposures of human populations to these pesticides, even when exposures were unsuspected (Whysner, 2020).

**FIGURE 2:**
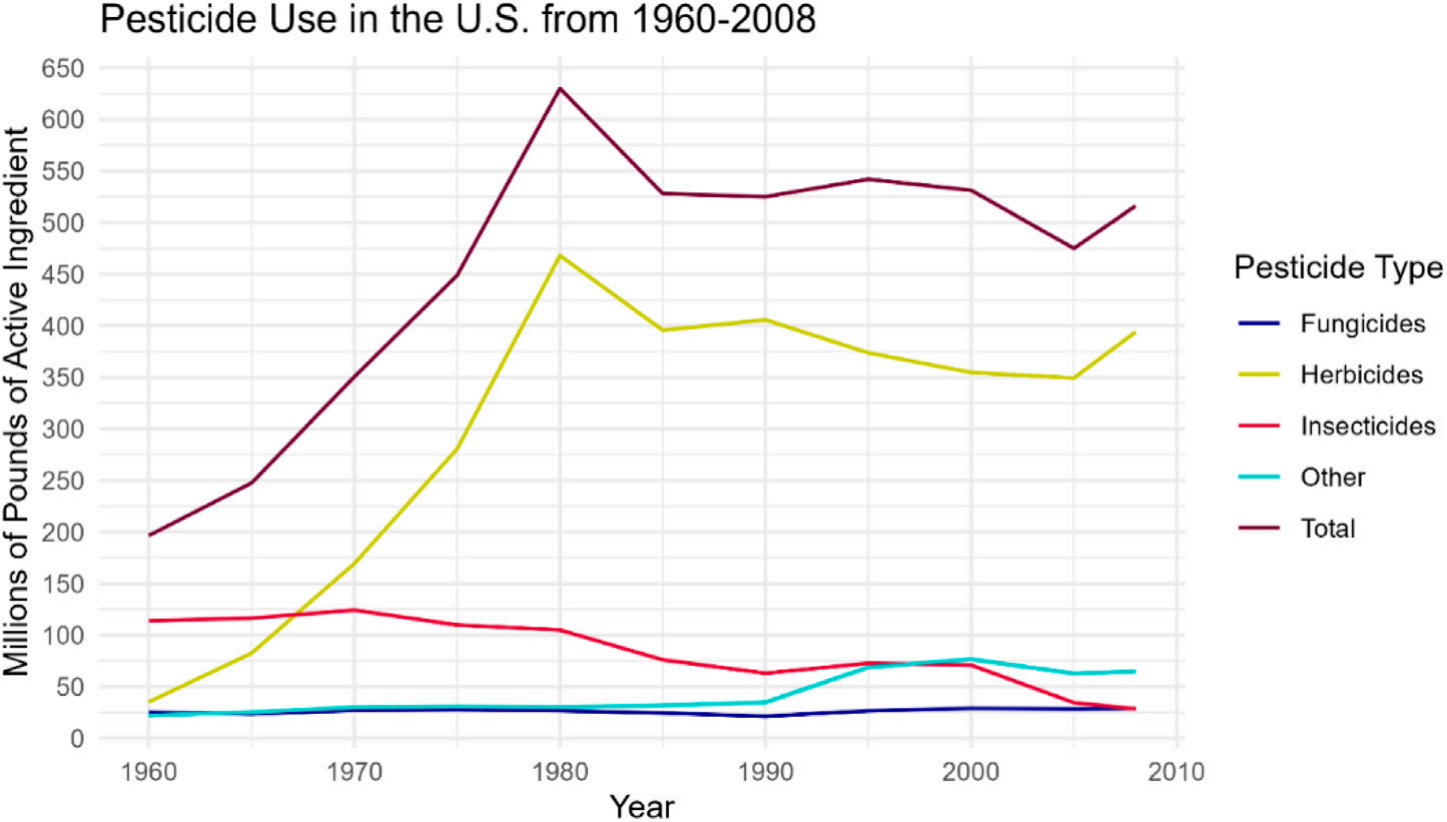
Changes in pesticide use in the US over time. Data from the US Department of Agriculture illustrates the drastic increase in the volume of pesticide active ingredients produced between the 1960s and the 1980s, and the relative levelling off of pesticide volume between the 1980s and 2008. Source of data: (Fernandez-Cornejo et al., 2014).

Between the 1960s and the 2000s, the use of pesticides for the production of crops changed drastically in the United States (Fernandez-Cornejo et al., 2014). When adjusted for inflation, expenditures on pesticides increased from approximately US $2B in 1960 to a peak of approximately US $15B in 1998, followed by a modest decline to a yearly expenditure of US $12B in 2008. The increase in expenditures was matched by an increase in the volume of pesticides used over roughly the same period of time (Figure 2). However, most of the growth in the volume of pesticides used was due to a striking increase in the volume of herbicides, from 16 million kilograms in 1960 to 179 million kilograms in 2008. Over the same period of time, insecticide use declined from 52 million kilograms in 1960 to 13 million kilograms in 2008.

As described earlier, global consumption of agricultural pesticides also rose significantly, especially between the two-decade period from 2001 to 2020. During this time, global pesticide consumption increased from 2.18 million metric tons to 3.45 million metric tons (a 58% increase; see Figure 1). Herbicide use doubled over these 2 decades (from 0.93 million metric tons to 1.86 million metric tons), whereas fungicide and biocide use increased by 44% and insecticide use increased by only 35%.

Trends in insecticide use, both in the US (which showed a decline over almost 5 decades) and globally (which showed a more modest use overall relative to other pesticides) may be attributed at least in part to increased resistance of target insect populations to the most popular insecticides, and the phasing out of the use of these chemicals as they are found to be significantly less effective (Fernandez-Cornejo, 1999). The striking increase in herbicides observed in both the US and globally can largely be attributed to the genetic engineering of herbicide-resistant crops (Fernandez-Cornejo et al., 2014). The creation of genetically modified herbicide-resistant crops led to large increases in the use of specific herbicides such as glyphosate, which is widely used in the production of genetically modified soybean, cotton and corn crops (Benbrook, 2018).

#### Pesticides have off-target effects

By the time *Silent spring* was published in 1962, debate about the hazards of pollutants had begun, and population-level effects of DDT on birds in natural ecosystems began to be documented and acknowledged. In the 1980s, a study conducted in Lake Apopka, Florida, United States followed the effects of pesticides on the American alligator (*Alligator mississippiensis*) after an accidental spill of DDT and dicofol, a pesticide used to control mites. 5 years after the spill, field studies found significant drops in the fertility success rates and high rates of mortality in large alligators (Woodward et al., 1993). Other studies documented an increase in the incidence of abnormalities of the reproductive system in hatchling and juvenile alligators including abnormal ovarian morphology in females and poorly organized testes in males (Guillette et al., 1994). The Nile crocodile (*Crocodylus niloticus*) has similarly been shown to be massively contaminated with DDT and other pesticides (chlordanes, cyclodeines), with measurable levels detected in muscle tissues of crocodiles in South Africa’s Kruger National Park reaching >150,000 ng/g lipid weight (Gerber et al., 2021). These levels are 120-times higher than had previously been reported in any other wildlife species, and there is strong suspicion that these pesticide exposures contributed to sudden mass deaths of hundreds of these animals within the park in 2008 (Ferreira and Pienaar, 2011).

Birds and humans were never intended to be exposed to DDT (or most other pesticides). Thus, the impact of these exposures on non-target species raises concern about the ability to control exposures to these pesticides once they are released into the environment. DDT was phased out of use in many countries not only because of the concerns for these chemicals on the health of people, wildlife and ecosystems, but also because of the development of resistant insects, rendering the insecticide ineffective in controlling malaria vectors like mosquitoes (Van den Berg, 2009; Vatandoost et al., 2022). Similar resistance has also been documented in weed species following use of herbicides such as glyphosate, with almost 50 weed species that are now known to have evolved resistant strains to this chemical (Baek et al., 2021).

Concerns have also been raised about the specificity of the compounds for their target species. Neonicotinoid pesticides (often referred to colloquially as “neo-nics”) provide an example of the consequences for non-target species following exposures due to shared molecular targets across species. Neonicotinoid pesticides were designed to target the nicotinic acetylcholine receptor (AChR), a ligand-gated cation channel found in cells including nerves; when the receptor is bound, ions are released uncontrolled, causing abnormal neuronal excitability, paralysis and death (Houchat et al., 2020). Neuronal AChRs are found in non-target insects (like honeybees and other pollinator species), mammals, and fish, raising concerns that these insecticides can impact non-target species in ecosystems and through the food chain. Neonicotinoids have been measured in freshwater at greater concentrations than other insecticides, and several studies have shown that they have toxic effects on aquatic organisms including both invertebrates and vertebrates (Malhotra et al., 2021). For example, a controlled study of freshwater shrimp revealed that exposures to even low concentrations (31 ppt) of the neonicotinoid pesticide imidacloprid reduced locomotion (up to full immobilization of the shrimp), reduced heart rate, decreased the rate of gill ventilation (e.g., breathing rate), and induced death (Siregar et al., 2021). Neonicotinoids also have highly acute toxic effects on wild honeybees, potentially contributing to declines in honeybee populations. Mass colony losses of honeybees have been documented in many countries during crop planting season, with dead bees containing high levels of neonicotinoid pesticides, providing evidence of an association between exposures and adverse outcomes (Starner and Goh, 2012).

Because of structural differences in the neuronal AChR between insects and mammals, neonicotinoid pesticides have been characterized as “poor activators” or weak agonists of mammalian neuronal AChR (Houchat et al., 2020). Yet, rodent studies have revealed effects of neonicotinoid pesticides on neurological outcomes such as altered learning, memory, and other behavioral traits, suggesting that these pesticides may utilize other modes of action in non-target species, contributing to adverse outcomes (Ongono et al., 2020).

#### Pesticides do not stay where they are used

Persistent organic pollutants (POPs) have the ability to move throughout ecosystems and trophic levels as well as be transported globally (Tanabe et al., 1997). For example, DDT and its metabolites are known POPs; the two major metabolites of DDT, dichlorodiphenyldichloroethylene (DDE) and dichlorodiphenyldichloroethane (DDD), are formed under anaerobic and aerobic conditions, respectively, via biotic (microbial conversion) or abiotic (chemical breakdown, photodegradation) processes (Bosch et al., 2015). These metabolites are more stable than DDT, with DDE having a particularly high environmental stability. DDT and its metabolites are hydrophobic and can be stored within the fat of both humans and wildlife (Liang et al., 2020); these chemicals persist in nature and biomagnify in the food web (Liang et al., 2020). In a study examining the atmosphere above a field where DDT was applied, 66% of the total applied pesticide was detected as p,p’-DDE (Cliath and Spencer, 1972), indicating that a majority of the DDT in soil is volatilized as the more stable DDE metabolite.

The volatility of pesticides increases their ability to be transported globally (Bartrons et al., 2016). Even though the use of many persistent pesticides has been restricted, these compounds are found in places where they are no longer used or were never used. For example, in a study conducted in the Canadian Rocky Mountains, high levels of organochlorine compounds were found in the snow at higher altitudes where there is greater precipitation (Blais et al., 1998). This result demonstrates that there is a potential for higher altitudes to retain semi volatile organochlorine pesticides where they were not produced or used and are otherwise not expected to be found.

Extraordinary levels of POPs have also been detected in two of the world’s deepest ocean trenches (Jamieson et al., 2017). Furthermore, these chemicals were found in the endemic amphipod fauna within these trenches, indicating that POPs are present even in the deepest, untouched parts of the ocean. Similarly, chlorinated pesticides have been detected in arctic snow, glacier ice and glacier melt (Pawlak et al., 2022; Pawlak et al., 2021), and in the bodies of people living in or near the Arctic circle (Long et al., 2021). Again, these results suggest that pesticides are detected far from where they were originally used.

#### Climate change can exacerbate pesticide use

Climate change has the potential to significantly shift the volume of pesticides used in agriculture as well as the use patterns for these pesticides. Warmer temperatures will increase the length of growing seasons and the number of months each year that pests are present (Skendžić et al., 2021). While climate change is impacting areas around the world in different ways, high temperatures and shifts in precipitation amounts resulting from climate change are causing the expansion of the geographic range of insects and other pests, increasing the spreading of invasive species. Warmer temperatures also allow some species of insects to survive during the winter months when historically they would be dormant, which will increase the number of generations produced annually.

Increasing global temperatures will also lead to the development of inhospitable conditions for important staple crops. For example, as a result of higher temperatures, mathematical modeling of crop growth data predicts that there will be northern migration of staple food crops (Tubiello et al., 2002), contributing to a global decline in wheat production of up to 16%, even with increased wheat production anticipated in many regions of Africa (Guo et al., 2024). Climate change is already affecting grape cultivation, and 90% of traditional wine grape growing areas are at risk of destruction by the year 2100, although new areas of grape farming are likely to be identified, shifting the use of pesticides to these locales in Tasmania, northern France, and the southern United Kingdom (van Leeuwen et al., 2024). Increasing temperatures could also lengthen growing seasons in northern latitudes, which could potentially lead to more months each year in which pesticides are applied in these geographic areas. Similarly, changes in precipitation patterns associated with climate change could influence pesticide run-off and unintended exposures of non-target species. For example, a worst-case scenario model for atrazine exposures in the Midwestern US corn belt calculated that increased precipitation attributed to climate change could cause an increase in atrazine migration, ultimately resulting in larger amounts of the pesticide reaching the groundwater table (Liu et al., 2022).

Although the impact of climate change on pesticide use will be complex and likely region-specific, it is also notable that pesticide production itself can contribute to climate change. Climate altering gases including carbon dioxide, methane, and nitrous oxide are emitted during the manufacture of pesticides (Heimpel et al., 2013).

Finally, higher temperatures can increase the volatilization of POP pesticides. This can lead to an increase in these pollutants’ release, mobilization, and degradation into the air, water, and soils (Teran et al., 2012). Thus, a changing climate is likely to increase the global transport of pesticides and other pollutants, depositing these chemicals far from where they were originally used.

### Pesticides are a global challenge to equity and justice

Public health scientists have demonstrated that there are inequities in health outcomes in human populations, making it critical to understand, consider, and address how social determinants of health contribute to such disparities (Baltruks et al., 2022). More recent analyses have examined how global factors are linked to specific health risks, and many of these risks are influenced by other social determinants of health including race and ethnicity, sex and gender, occupation, socioeconomic status and income (Kemarau et al., 2024).

**FIGURE 3:**
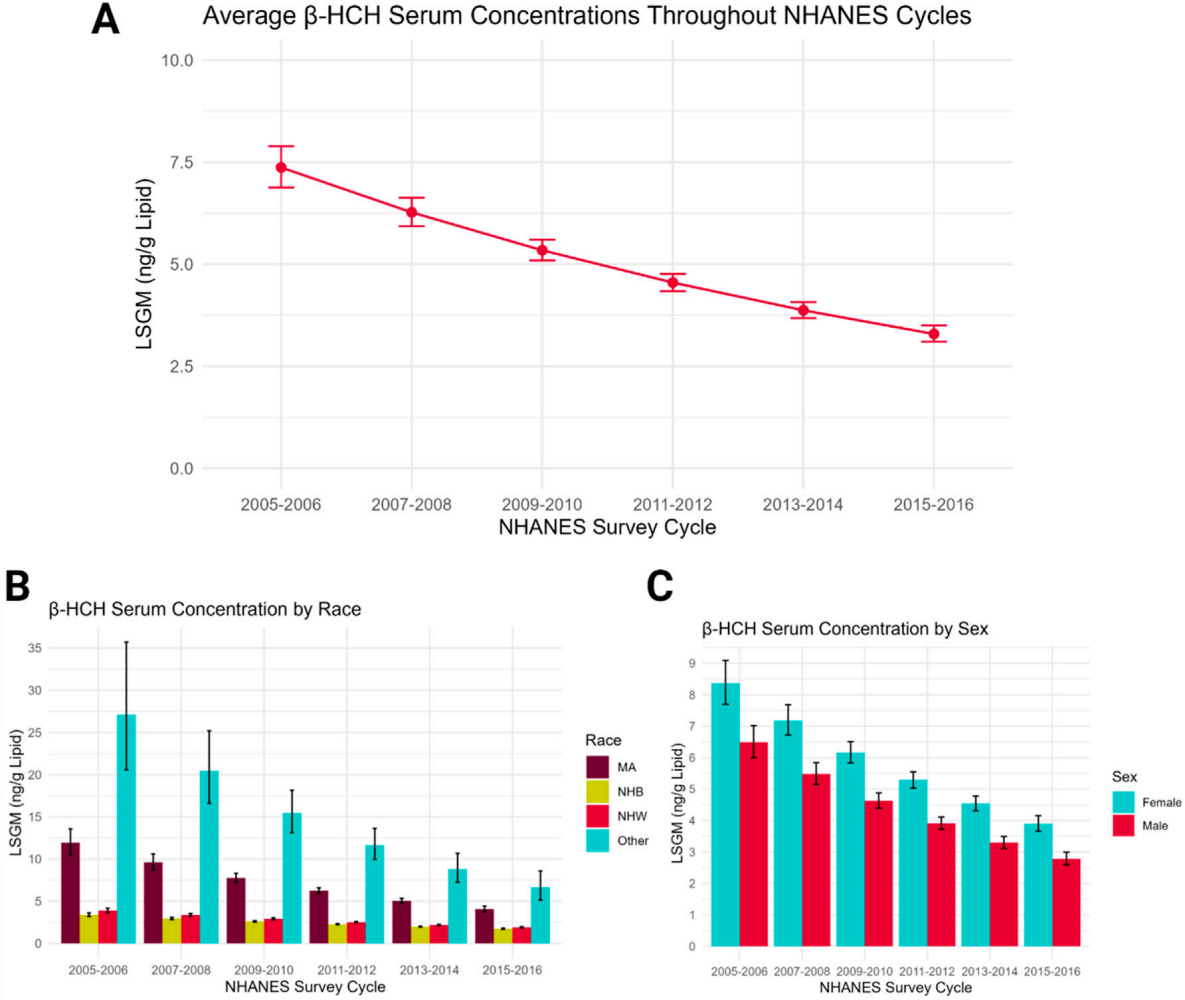
Serum concentrations of β-HCH point to social determinants impacting exposures to this persistent pesticide. **(A)** Concentrations of β-HCH measured in the general population declined in the 10 year period between 2005 and 2015, but remained above 3 ng/g lipid even 30 years after production had ceased and use had been banned in the US. **(B)** Comparisons across racial and ethnic groups reveal that exposures are highest in the “other” category, consisting of Indigenous, Asian, and non-Mexican Hispanic populations. Exposures are also significantly higher in Mexican American (MA) compared to Non-Hispanic Black (NHB) and Non-Hispanic White (NHW) populations. **(C)** Comparisons across sexes indicate that females have consistently higher concentrations of β-HCH detected in serum. In all panels, concentrations are reported as least squares geometric means (LSGM), a measure that represents the central tendency of each group. Data from this figure come from (Li et al., 2022).

Organochlorine pesticides (OCPs) are a class of pesticides that are highly persistent in the environment and have been linked to altered hormone action, neurotoxicity, cancer, and liver and kidney damage (Jayaraj et al., 2016). As a result, OCPs were banned from use in the United States and most other high-income nations in the 1970s and 1980s. Beta hexachlorocyclohexane (β-HCH) is the most chemically and physically stable isomer of the insecticide lindane, making it highly resistant to degradation in the environment (Rubini et al., 2020). β-HCH has a half-life in humans of about 7 years and can bioaccumulate in lipids, so exposures can last long beyond the direct use and production of this chemical. Despite being banned for use in the United States in the 1980s, β-HCH was still found at detectable levels in the general population 30 years later, although concentrations continue to decline with time (Figure 3A). A study based on NHANES data found an average serum concentration of 3.29 ng/g lipid β-HCH detected in people in the U.S. in the 2015–2016 cycle (Li et al., 2022). β-HCH is an example of a pesticide with disproportionate exposures across general human populations; in the US, levels of β-HCH were consistently twice as high in Mexican Americans compared to non-Hispanic white and non-Hispanic Black populations (Figure 3B). For all ethnicities other than Mexican American, non-Hispanic white, and non-Hispanic Black (i.e., the “other” category includes Indigenous, Asian, and non-Mexican Hispanic populations), exposure was three times as high as white Americans. The disproportionate exposures in marginalized racial groups may contribute to other health disparities.

Sex also appears to be an important factor influencing exposures (Figure 3C). A study in Italy of a population surrounding a chemical dumping site with high levels of β-HCH contamination found consistently higher serum concentration in females compared to males, even when adjusting for confounding factors such as age (Narduzzi et al., 2020). Whether women are exposed at greater rates or metabolism and excretion rates are slower in females, a disproportionate impact on women is cause for concern, especially because β-HCH, like other OCPs, can cross the placental barrier and be passed to offspring through breastmilk. Higher pesticide exposures in pregnant women could have detrimental impacts on these women directly, as well as on fetal development.

Country-specific usage of pesticides is also a critically important social determinant of pesticide exposure. Brazil has one of the highest levels of pesticide use, accounting for 800,650 metric tons in the year 2022, accounting for almost a quarter of all pesticides applied across the globe (Faostat Analytical Brief, 2022). Approximately one-third of all pesticides used in Brazil have been banned from use in the EU, and the maximum residue limits allowable for others can be 400-times higher in Brazil compared to the EU. These striking disparities in pesticide use between the global north and the global south may exacerbate other environmental vulnerabilities that have been observed in countries like Brazil (Perobelli, 2025).

Occupation is perhaps the most critical social determinant of pesticide exposure. Agricultural workers bear the brunt of pesticide exposure as these individuals apply the chemicals and directly handle crops where pesticides have been used. Mexican immigrants make up 69% of the population that is tasked with directly handling pesticides in the US (Mehta et al., 2000). Exposures to farmworkers occur through the oral, dermal and inhalation routes, and are documented in pesticide applicators as well as crop pickers (EPA). Migrant farmworkers have some of the worst documented health outcomes in the US, and although these outcomes are not due solely to occupational chemical exposures, the contribution of pesticide exposures cannot be discounted (McCauley et al., 2001).

The disproportionate exposure of farm workers to pesticides also carries over to their families. The “occupational take-home pathway” of pesticide exposure means that households of farmworkers have greater concentrations of pesticides in house dust compared to non-farmworker houses (Bennett et al., 2019). Pesticides can linger on clothing and other personal belongings that travel to work with the workers, and since these individuals often live close to where they work, pesticides can enter their homes via air (McCauley et al., 2001). The implications of increased exposure to pesticides for children in the house cannot be understated, and the inequities in exposure based on job status, occupation, and other socioeconomic factors influencing the family adds another layer of injustice to the global health threat of pesticides.

Despite their negative impact on the environment and human health, the use of OCPs is increasing worldwide as low-income countries continue to use OCP insecticides such as DDT and β-HCH. Although the use of DDT for agricultural uses was banned or heavily restricted in most high-income countries starting in the 1970s and 1980s, in 2006 the World Health Organization advocated for the spraying of DDT in some low- and mid-income countries for the control of malaria-carrying mosquito populations (Organization, 2006). Furthermore, studies of agrarian countries like Ethiopia reveal that DDT is not only used as vector control for malaria, it also continues to be used for agricultural purposes (Negatu et al., 2021; Debela et al., 2023), contributing to high DDT exposures of wildlife (Yohannes et al., 2013), measurable residues in staple crops (Mekonen et al., 2014) and dairy (Deti et al., 2014), and in human biomonitoring samples, including in breast milk (Gebremichael et al., 2013).

These findings emphasize an issue of inequity surrounding global regulation and continued use of pesticides. While regulation of pesticides is critical for reducing exposure and the burden of harm on human health, banning or restricting the use of a pesticide in high-income jurisdictions is not a comprehensive solution to exposure if usage continues in other parts of the world.

### The planetary boundaries framework provides a global view of the impact of pesticides

The concept of planetary boundaries was introduced more than a decade ago as a framework to understand and evaluate how human actions impact various earth systems, and whether such actions prevent the earth’s biophysical systems from being maintained sustainably (Rockström et al., 2009). As a part of this framework, scientists from across disciplines worked to establish “safe operating spaces” for humans and their activities’ impact on the ecological systems of the earth. This initial work arose from a shared understanding that human activities were having significant, and sometimes irreversible effects, on key aspects of environmental health.

**FIGURE 4:**
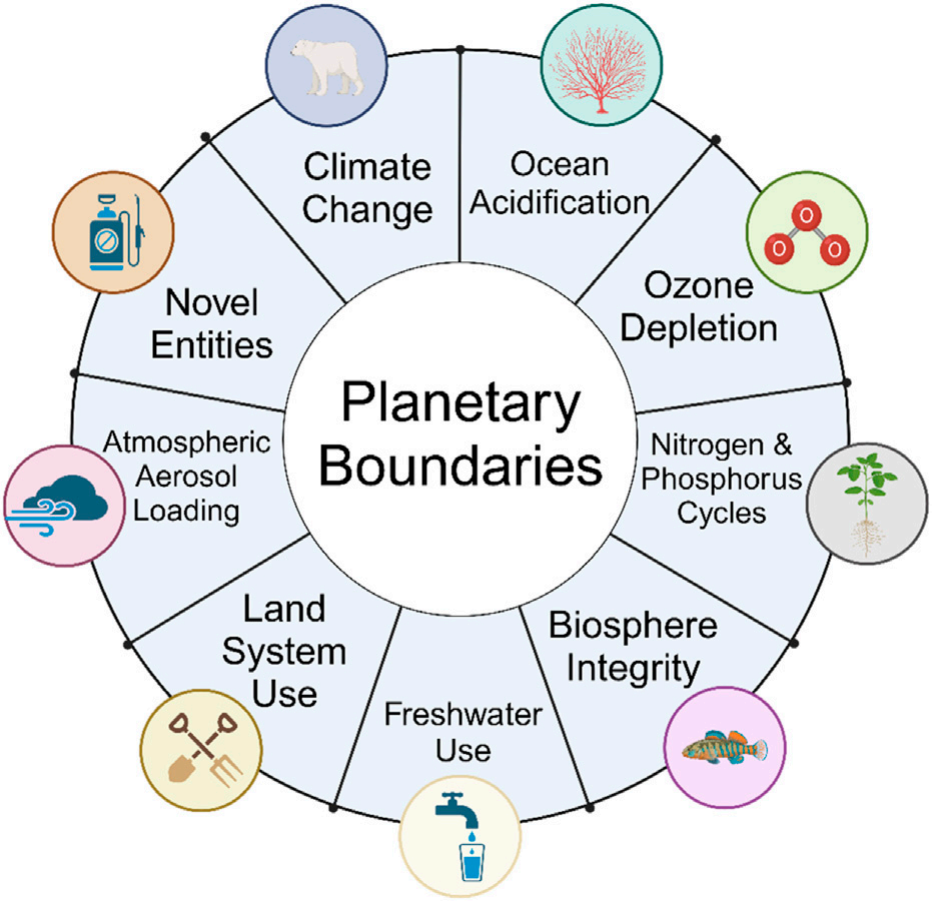
The nine planetary boundaries. Pesticide use can impact nitrogen and phosphorus cycles, biosphere integrity, the availability of freshwater, land-system use, and atmospheric aerosol loading. However, the greatest challenge posed by pesticide use is the “novel entities” boundary because pesticides have an increasing rate of production, increasing rates of release into the environment, and diverse risk potentials that exceed the ability of society to conduct safety assessments, monitor exposures, and adequately evaluate their effects (Persson et al., 2022).

Thus, planetary boundaries (sometimes referred to as Earth System Indicators) became a concept for addressing global challenges from the perspective of sustainability. Nine boundaries were proposed (Steffen et al., 2015) (Figure 4):

• climate change (including carbon dioxide in the atmosphere as well as measures of global temperature),
• ocean acidification (due to CO_2_ absorption, and effects on marine life),
• ozone depletion (including thinning of the ozone layer in localized places),
• alterations to nitrogen and phosphorous cycles (and other biogeochemical flows),
• biosphere integrity (including loss of biodiversity, and the rate of species extinction),
• freshwater use (due to the withdrawal of freshwater from ecosystems),
• land-system use (including deforestation and land conversion for agricultural purposes),
• atmospheric aerosol loading (such as the release of particulate matter into air, which affects both air quality and other measures of climate health),
• and novel entities (which includes the introduction of synthetic chemicals and other materials into the environment).

Although this framework continues to be updated from its original conception, and new information contributes to the understanding of the threshold for each planetary boundary, the concept has become adopted across numerous fields that focus on sustainability (Kemarau et al., 2024). Global discussions on planetary boundaries recognize that human activities that push beyond the threshold for one or more of these boundaries could lead to instability of the planet’s health, and ultimately put the survival of both ecosystems and human societies at risk.

Importantly, longitudinal evaluations of the nine planetary boundaries suggest that these continue to be transgressed, and that the situation has become more dire with time. Table 2 summarizes the evolution of the framework and the assessment of the thresholds for each boundary in 2009, 2015, and most recently in 2023 (from (Kemarau et al., 2024)). The continued assessment of planetary boundaries revealed emerging evidence that several boundaries have been crossed.

**TABLE 2:** Transgression of the planetary boundaries from 2009 to 2023.

Boundary	2009	2015	2023
climate change	C	C	C
ocean acidification			
ozone depletion			
alterations to nitrogen and phosphorous cycles	C	C	C
biosphere integrity	C	C	C
freshwater use			C
land-system use		C	C
atmospheric aerosol loading	NYQ	NYQ	
novel entities	NYQ	NYQ	C

C, crossed, NYQ, not yet quantified.

There is strong evidence that pesticides are impacting ecosystems, and can contribute towards pushing several planetary boundaries past their established thresholds of safe operations. Significant work has examined the impact of pesticides (on their own, or in concert with other agrochemicals) on biogeochemical cycles. First, phosphorus extraction is essential for the production of numerous pesticides (Yuan et al., 2018) and concerns have been raised that the planet’s reserves are insufficient to support continued and growing needs. Furthermore, phosphorus reserves are not distributed evenly around the planet, leading to disruptive extraction practices in localized places (especially in the Sahara region), and the processing of phosphate rock can release radioactive materials and heavy metals into the environment (De Boer et al., 2019). Also relevant to biogeochemical cycles is the impact of pesticides on the nitrogen cycle including microbial composition and activities of microbes (e.g., respiration, enzymatic activity, and ultimately nitrogen fixation). There is increasing evidence that several herbicides are toxic to nitrifying and nitrogen-fixing bacteria, and their presence can compromise this aspect of soil fertility (Brochado et al., 2023).

Pesticide use also has demonstrated impacts on biosphere integrity, including loss of biodiversity. As described previously, many pesticides are known or suspected EDCs (Demeneix, 2020) and reproductive and developmental toxicants, and have been shown to be associated with declines in the populations of wildlife in many different ecosystems (Gore et al., 2015; Crain et al., 2008; Guillette, 2006). Numerous studies have focused on whether pesticides, and especially neonicotinoid pesticides, might be contributing to the deaths of non-target species including pollinator insects (Brittain et al., 2010), freshwater invertebrates (Beketov et al., 2013), and other species that are critical to the health and function of ecosystems.

Certainly, there is also evidence that pesticide use can impact the availability of freshwater, considering the extensive evidence that these chemicals contaminate drinking water supplies (Syafrudin et al., 2021). Pesticides can enter drinking water through both agricultural run-off and production processes. In the US, evaluations conducted between 1992 and 2001 by the Department of the Interior and the US Geological Survey revealed that pesticides were detected in more than 90% of all streams, and in more than 25% of all aquifers (Gilliom et al., 2006). Similar contaminations have been observed in drinking water sources such as the Tengi river in Malaysia, where concentrations of the insecticide imidacloprid were reported as high as 60 ppb, and the fungicide tebuconazole were as high as 510 ppb (Elfikrie et al., 2020); the Shinano River in Japan where detectable levels of 22 herbicides, 15 insecticides, and 11 fungicides were reported, with the highest levels exceeding 8,000 ppb for the fungicide isoprothiolane (Tanabe et al., 2001); and the Dongjiang River in China, where the presence of pesticides commonly used in the surrounding agricultural region was reported, with concentrations of this same fungicide found above 250 ppb (Zhang et al., 2020). These studies highlight that the contamination of drinking water sources is a global concern.

Of course, the greatest challenge posed by pesticide use is the “novel entities” boundary, which focuses specifically on the introduction of synthetic chemicals and materials into the environment. Although pesticides are only estimated to account for ∼2% of all synthetic chemicals made globally (Alavanja, 2009), because these chemicals are designed to be biologically active, their disproportionate effects raise significant concern for the health of people and the environment. The release of pesticides and other synthetic chemicals into the environment is one of the fastest growing challenges to planetary boundaries, with a greater rate of change than other agents challenging sustainability including release of carbon dioxide into the atmosphere (Shattuck, 2021). Unfortunately, even organizations dedicated to studying global agents of change have given little attention to the challenges posed by synthetic chemicals to planetary health, sustainability, and the resilience of the planet to human activity (Bernhardt et al., 2017).

## Conclusion

In the 1960s, Sir Austin Bradford Hill assembled a series of nine ‘viewpoints’, e.g., criteria that could be used in observational studies to help build causal arguments between environmental agents and adverse health effects (Hill, 1965). Much has been written about whether these are the correct criteria and whether they work for all environmental pollutants (Zoeller et al., 2014). While a whole field of study has been created around how to demonstrate causal relationships in observational epidemiology studies, much less attention has been given to Bradford Hill’s argument that the consequences of *inaction* need to be weighed when evaluating the potential effects of an environmental agent. He wrote, “on relatively slight evidence we might decide to restrict the use of a drug for early morning sickness in pregnant women. If we are wrong in deducing causation from association no great harm will be done … On fair evidence we might take action on what appears to be an occupational hazard, e.g., we might change from a probably carcinogenic oil to a noncarcinogenic oil in a limited environment and without too much injustice if we are wrong” (Hill, 1965). These words, also consistent with the precautionary principle (Tickner, 2004), reflect what is needed in the evaluation of pesticides and their impact on humans, wildlife, and planetary health: even in the face of some uncertainties, if there is the possibility of catastrophic damage associated with pesticides, regulators and other decision-makers should restrict their use (Drivdal and van der Sluijs, 2021).

Certainly, when it comes to the planetary boundaries, catastrophic damage to both ecosystems and human populations is anticipated when a boundary is transgressed. Experts have determined that the production of synthetic chemicals has already exceeded the novel entities planetary boundary, and pesticides are a part of this global challenge.

Importantly, the planetary boundaries framework is useful to push scientists and decision-makers towards actions that reduce both the production and the use of chemicals, like pesticides, that contribute to the transgression of the boundary. This is especially critical considering estimates that only 1% of all pesticides that are applied target their intended pests (Perobelli, 2025). There are alternative approaches that utilize integrated pest and pesticide management strategies (Peshin and Zhang, 2014), and these approaches can contribute to significantly lower volumes of pesticides, as well as a shift to less toxic pesticides (Barzman et al., 2015).

Pesticides have become a feature of modern living, useful in the control of weeds, insects, and other pests. Many successes have been attributed to these chemicals including the production of crops and the control of insects and other vermin known to spread infectious diseases. Over several decades, new classes of pesticides have been introduced, often with the goal of replacing more toxic chemicals with less toxic alternatives. Despite these advances, the continued use of pesticides raises concerns about the challenges posed by these chemicals to the individuals who are most heavily exposed (e.g., occupational users), non-target species (including humans and wildlife), and more generally the challenges posed to global health and planetary boundaries. Addressing the impact of pesticides and the underlying treadmill of production that props up their continued use, even when their efficacy is challenged and the health of humans and the planet are put at risk, is a public health crisis. Efforts are urgently needed to address the risks that pesticides pose to planetary health.

## Abbreviations

AChR, acetylcholine receptor; DDD, dichlorodiphenyldichloroethane; DDE, dichlorodiphenyldichloroethylene; DDT, dichlorodiphenyltrichloroethane; EDSP, endocrine disruptor screening program; HCH, hexachlorocyclohexane; MA, Mexican American; NHB, Non-Hispanic Black; NHW, Non-Hispanic White; NOAEL, no observed adverse effect level; OCP, organochlorine pesticides; POPs, persistent organic pollutants.
